# Biomarkers for response in major depression: comparing paroxetine and venlafaxine from two randomised placebo-controlled clinical studies

**DOI:** 10.1038/s41398-019-0521-7

**Published:** 2019-08-02

**Authors:** Lucia Carboni, Dennis J. McCarthy, Bruno Delafont, Michele Filosi, Elena Ivanchenko, Emiliangelo Ratti, Susan M. Learned, Robert Alexander, Enrico Domenici

**Affiliations:** 1 Department of Pharmacy and Biotechnology, Alma Mater Studiorum University of Bologna, Bologna, Italy; 2 Indipendent Consultant, Clinical Pharmacology and Translational Science, Newark, DE USA; 3 Delafont Statistics, Alençon, France; 4 Department of Cellular, Computational and Integrative Biology, University of Trento, Trento, Italy; 5 Leadiant GmbH, München, Germany; 6 Neuroscience Therapeutic Area Unit, Takeda, Boston, MA USA; 7 Global Medicines Development, Indivior, Inc., Richmond, VA USA; 8 Fondazione The Microsoft Research-University of Trento Centre for Computational and Systems Biology (COSBI), Rovereto, TN Italy

**Keywords:** Predictive markers, Neuroscience, Depression

## Abstract

The identification of biomarkers of response might speed drug development and set the premises to assist clinical practice in psychiatry. In this work, we evaluated a panel of peripheral biomarkers (including IL-6, IL-10, TNF-α, TNFRII, BDNF, CRP, MMP9 and PAI1) in depressed patients receiving paroxetine, venlafaxine, or placebo. Samples were obtained from two randomised placebo-controlled studies evaluating the efficacy and tolerability of a novel drug candidate, using either paroxetine or venlafaxine as active comparators. In both studies, the biomarker candidates were analysed in plasma collected at randomization and after 10 weeks of treatment with either placebo or active comparator (for a total of 106 and 108 subjects in the paroxetine and venlafaxine study, respectively). Data were obtained by multiplexing sandwich-ELISA system. Data were subjected to statistical analysis to assess their correlation with baseline severity and with response outcome. Increases in biomarker levels were correlated with reduction in depression severity for TNF-α, IL-6 IL-10 and CRP. Response to paroxetine treatment correlated with baseline IL-10, IL-6 and TNF-α levels, with the strongest signal being observed in males. In the venlafaxine study, a correlation was observed only between CRP level at randomisation and response, suggesting differences between the two active treatments and the two studies. Our investigations suggest that a combination of pro- and anti-inflammatory cytokines may predict response outcome in patients treated with paroxetine. The potential for IL-10, IL-6 and TNF-α as response biomarkers for a wider range of antidepressants warrants further investigations in clinical trials with other monoamine reuptake inhibitors.

## Introduction

The application of biomarkers in drug development and clinical decision-making has the potential to change the delivery of healthcare dramatically. Biomarkers could have a profound impact on the diagnosis and treatment of psychiatric disorders, such as major depressive disorder (MDD)^1–3^. MDD is a severe psychiatric disorder with lifetime prevalence in excess of 15%, which is the fourth leading cause of disability worldwide^4^. Notwithstanding extensive biological research, the pathophysiology of depression remains elusive. At present, the diagnosis and treatment of MDD is still based on the subjective assessment of symptoms. Biomarkers in MDD could help identify homogeneous sets of patients who will benefit most from a particular treatment. Biomarkers could also complement clinical assessment by highlighting changes in the levels of biomarkers that occur in parallel or ahead of changes in clinical symptoms, allowing physicians to make adjustments in therapy quickly. Identification of biomarkers for likely placebo responders could provide a means to reduce the size (and therefore costs) of pivotal clinical studies. Finally, biomarkers could eventually lead to the selection of more efficacious and tolerated treatments in clinical practice^5^.

A number of biological hypotheses have been exploited in the search for MDD biomarkers. The immuno-inflammatory hypothesis of MDD is based on the discovery of reciprocal communication between the immune and nervous systems^6–8^. It is known that inflammatory stimuli can elicit depressive-like symptoms both in preclinical species and in humans, and several data indicate an altered immunologic state in depression. For example, pro-inflammatory cytokines and bacterial endotoxins elicit sickness behaviours and symptoms observed in anxiety and depressive disorders that can be attenuated by chronic antidepressant treatment^8^. Similarly, the therapeutic use of IL-2 or INF-α induces depressive symptoms that are similar to those seen in MDD. In addition, it has been demonstrated that anti-cytokine treatment in chronic inflammatory conditions leads to improved depressive symptoms^9^. The potential application of anti-inflammatory agents in depression was tested in a proof-of-principle study with a TNF antagonist which showed that while TNF antagonism did not exhibit generalized efficacy, it improved depressive symptoms in a subgroup of patients with increased inflammation^10^. Accumulating evidence supports the hypothesis that peripheral immune activation plays a role in the pathophysiology of MDD, as documented by many studies observing altered levels of cytokines, chemokines and other inflammatory markers in depressed patients^11–15^. Data also show that antidepressant treatment may reduce peripheral inflammatory markers^16–20^. In particular, it has been investigated whether components of the inflammatory response could be used as predictive biomarkers able to direct or aid the selection of the best antidepressant agent. It has been proposed that elevated levels of inflammatory markers contribute to treatment resistance^18,21–26^. However, other studies did not consistently detect this increase and a recent meta-analysis could not detect statistically significant differences in cytokine levels between treatment responders and non-responders^20,27^. It should be remarked that a large degree of heterogeneity is evident in this literature, and that the above biomarkers might be affected by the concomitant presence of inflammatory conditions, or concomitant anti-inflammatory treatments, in MDD patients. Moreover, only a small number of studies investigated biomarkers of prognostic efficacy^20,28^.

We investigated the role of cytokines as biomarkers for response in MDD using data from two independent randomized, placebo- and active-controlled studies^29^. We selected protein markers (Supplementary Table 1) based on a biomarker study in depressed patients using a multiple analyte panel^30^ and supplemented by immuno-inflammatory markers derived from the literature. Six of the markers belong to immuno-inflammatory pathways, i.e., IL-6, TNF-α, TNFRII, IL-10, CRP and MMP9. This study addressed three questions: First, we investigated the association between pre-treatment biomarker levels and MDD severity, with the aim of identifying state markers for depression. Second, we analysed the association between changes in blood biomarkers and changes in depressive symptomatology, with the aim of identification markers for treatment efficacy. Third, we investigated the association between baseline biomarker profile and changes in efficacy endpoint, with the aim of identifying biomarkers that can predict treatment response to antidepressants.

## Methods

### Clinical studies

Studies SND103288 and SND103285 were clinical trials which compared the efficacy and safety of the “triple reuptake inhibitor” GSK372475 vs. a selective serotonin reuptake inhibitor (SND103288, hereafter “paroxetine study”, clinicaltrial.gov identifier NCT00448058) or a serotonin-norepinephrine reuptake inhibitor (SND103285, hereafter “venlafaxine study”, clinicaltrial.gov identifier NCT0042064) and placebo^29^. The results on efficacy, safety and tolerability have been described elsewhere^29^. Of note, no efficacy was seen with GSK372475, while the paroxetine and venlafaxine groups showed improvement on all efficacy measures relative to placebo^29^.

The studies were 10-week randomized, multi-centre, double-blind, parallel-group, placebo- and active-controlled, flexible-dose studies in male and female outpatients (18–64 years of age) with a psychiatric diagnosis of a major depressive episode associated with major depressive disorder according to DSM-IV-TR. The paroxetine study population consisted of 493 male and female subjects (mean age 42.9 ± 11.19, 70% females); 171 of them were randomized to GSK372475, 166 to paroxetine and 156 to placebo treatment group. 343 (70%) subjects completed the trial. The venlafaxine study population consisted of 393 male and female subjects (mean age 42.6 ± 11.67, 62% females); 134 of them were randomized to GSK372475, 133 to venlafaxine and 126 to placebo. 233 (59%) subjects completed the trial. For both studies, treatment efficacy was assessed by trained clinicians using the Inventory of Depressive Symptomatology (IDS), the 17-item version of the Hamilton Rating Scale for Depression (HAMD) and the 6-item Bech subscale derived from the HAMD. For the paroxetine study only, the Montgomery Asberg Depression Rating Scale (MADRS) was additionally used to assess treatment efficacy.

Both multi-centre study protocols were reviewed and approved by national, regional, or investigational centre Ethics Committees or Institutional Review Boards, and were conducted in accordance with the International Conference on Harmonisation (ICH) Good Clinical Practice guidelines, applicable country-specific requirements, and ethical principles outlined in the World Medical Association Declaration of Helsinki on the Ethical Principles for Medical Research Involving Human Subjects. Written informed consent was obtained from all subjects before their involvement in any study-related procedure.

### Patient selection

Since no efficacy was seen with GSK372475, the biomarker analysis was restricted to the paroxetine, venlafaxine and placebo groups. We applied a number of additional *a priori* criteria to limit the potential heterogeneity of the study population, as follows. Blood samples were submitted to biomarker analysis for patients: (i) of Caucasian origin; (ii) completing the week 10 assessment; (iii) not coded as protocol violators; iv) from centres where at least 10 patients completed the study. While these additional criteria may have impacted the generalizability of the findings, we felt that they were needed to minimize heterogeneity in biomarkers data (which might be sensitive to ethnicity and country-specific effects) and treatment compliance, thus increasing the chance of detecting biomarker-response associations.

### Measurement of biomarkers

Circulating biomarkers were analysed in blood samples collected at randomization (week 0), and week 10.

Approximately 2 ml of blood for biomarker analysis were obtained from a forearm vein at the study site. Blood samples were collected into tubes containing EDTA, immediately chilled on crushed water ice. Plasma was separated by refrigerated centrifugation (4 °C, 1000×*g* for 15 min) within 1 h of collection. The resultant plasma samples were removed, transferred to an EDTA tube and stored frozen at −20 °C pending shipment for analysis. Plasma profile for the selected protein panel of candidates (IL-6, IL-10, TNF-α, TNFRII, BDNF, CRP, MMP9, PAI1) was obtained by multiplexing sandwich-ELISA system based on chemiluminescent detection at Aushon, Inc. Single samples were randomized by GSK statisticians and shipped to Aushon, Inc. Each assay consisted of a 96-well plate custom arrayed with target protein-specific anti-human antibodies, a lyophilized recombinant standard for each assay, sample diluent containing 0.1% sodium azide, biotinylated antibody reagent, streptavidin-horseradish peroxidase (SA-HRP) reagent, SuperSignal stable peroxide solution, and SuperSignal luminol/enhancer solution and Wash Buffer. Plates were read using the SearchLight Black Ice Cooled CCD Camera System serial #200201. Images were analysed using Array Analyst software, and data analysis were completed and summarized using Microsoft® Excel. Diluted samples, standards and controls were incubated for one hour on the arrayed plates. All incubations were performed at room temperature with shaking at 200 rpm. Plates were decanted and washed six times before adding a cocktail of biotinylated detection antibodies to each well. After incubating with detection antibodies for 30 min, plates were washed three times and incubated for 30 min with SA-HRP. Plates were again washed before adding SuperSignal Femto Chemiluminescent substrate. The plates were immediately imaged using the SearchLight Black Ice imaging system, and data was analysed using Array Analyst Software. Concentrations of all unknown samples were back-calculated using results extrapolated from the corresponding standard curve and expressed as pg/ml blood volume. A four-parameter curve fit was used for all standard curves. A concentrated lyophilized standard for each protein analysed was reconstituted each day to prepare the standard curve. The top standard for each array was prepared by adding an equal volume of each reconstituted standard included in the array and sample diluent to a final concentration of 1:16 of the lyophilized vial concentration. The top standard was serially diluted 1:2, then 1:4 four times, and then 1:2. The assay standard curve range was selected to optimize sensitivity and linearity. Standard curve precision profiles and control performances are included in Supplementary Table 2. The control range was established by calculating the mean for two levels of controls and subtracting two standard deviations from the mean for the minimum level and adding two standard deviations to the mean for the maximum level. The lower limit of quantification (LQ) was defined as the lowest standard of the standard curve with discrimination of two standard deviations compared to the zero (see assay performance and lower limit of quantification in Supplementary Table 2). Samples falling below LQ were labelled as < BLQ (below limit of quantification).

### Statistical analysis

The distribution of each biomarker was explored. In order to achieve normality on the analysis scale, all markers were log-transformed prior to inclusion in statistical models. In order to have a more quantitative support to our normalization strategy, we have conducted a normality test using Shapiro’s test for all data before and after log normalization, (see Supplementary Fig. 1). Even though the analysis shows that after log transformation 5 out of 8 analytes still display a non-normal distribution according to the test, data skewness is significantly reduced, (as shown for example by the QQ plot for IL-10), providing enough confidence for a stable estimation of the parameters.

Therefore, changes from baseline in each marker were derived as changes in the log-values, and baseline covariates were included on the log-scale.

Bivariate mixed model analyses were used to assess the correlation between HAMD and each marker at baseline and at week 10, including biomarker and HAMD as dependent variables, with gender and centre as fixed effects.

Bivariate mixed model analyses were also used to assess the correlation between changes in HAMD and changes in each marker at week 10, including change in biomarker and change in HAMD as dependent variables, and adjusted for gender, biomarker baseline and HAMD baseline. The same bivariate analyses were also run for males and females separately, using the same model without gender as fixed effect.

The association between biomarker baseline values and changes in HAMD was estimated using analyses of covariance accounting for the effects of HAMD baseline value, gender and centre, as well as biomarker baseline and treatment by biomarker baseline interaction.

In order to assess the potential of biomarker baseline values as predictor, we conducted additional statistical analyses based on binary classification, i.e. responders vs. non-responders. The statistical significance of the differences in baseline levels of all markers according with response status and gender was measured by logistic regression or Wilcoxon Test. In order to provide an estimate of the diagnostic accuracy of each marker as response predictor in the paroxetine study, a Receiving Operator Characteristics (ROC) curve analysis was performed. Furthermore, a PLS-DA analysis was performed to assess the accuracy of the marker panel as a classifier.

All testing was done at a nominal significance level of 0.05. Statistical analyses were performed using the SAS software (SAS Institute, Cary NC) and R 3.5.3. For data visualisation and data mining the Spotfire software (TIBCO) was used.

## Results

### Study population characteristics

The selection of the patients in which biomarkers were measured, resulted in a subset of 106 male (32%) and female (68%) patients from the paroxetine study (Table 1a) and 104 male (38%) and female (62%) patients from the venlafaxine study (Table 1b).

**Table 1a:** Baseline demographic and clinical data of biomarker population in the paroxetine study (SND103288)

		Biomarker population		
		Paroxetine (*N* = 52)	Placebo (*N* = 54)	Total (*N* = 106)
Age	Mean	45.88	46.35	46.12
	SD	9.99	9.69	9.84
	Min	22	21	21
	Max	63	63	63
Sex	Female	33 (63%)	39 (72%)	72 (68%)
	Male	19 (37%)	15 (28%)	34 (32%)
HAM-D	Mean	21.67	23.40	22.56
	SD	3.84	4.34	4.19
	Min	13	10	10
	Max	32	32	32

**Table 1b:** Baseline demographic and clinical data of biomarker population in the venlafaxine study (SND103285)

		Biomarker population		
		Venlafaxine (*N* = 51)	Placebo (*N* = 53)	Total (*N* = 104)
Age	Mean	44.80	44.53	44.66
	SD	11.10	10.46	10.78
	Min	21	19	19
	Max	63	60	63
Sex	Female	29 (57%)	35 (66%)	64 (62%)
	Male	22 (43%)	18 (34%)	40 (38%)
HAM-D	Mean	23.62	24.45	24.05
	SD	4.16	4.20	4.20
	Min	16	16	16
	Max	34	33	34

The biomarker population was compared with the “intention to treat” (ITT) population of the SND103288 and SND103285 studies (Supplementary Table 3), to estimate the potential presence of a selection bias given that the randomization of the ITT is lost in the biomarker population. The slight differences in mean age between ITT and biomarker populations (46.1 years vs. 43.1 in the paroxetine study and 44.7 vs. 42.4 in the venlafaxine study, Supplementary Table 3) were not considered as clinically meaningful, although statistically significant. At randomisation (baseline), the severity of depression as assessed by HAMD did not significantly differ among the paroxetine, venlafaxine and placebo groups in both studies.

The active comparators paroxetine (study SND103288) and venlafaxine (study SND103285) demonstrated superiority compared with placebo across all primary and secondary efficacy endpoints (*p* < 0.001)^29^.

We then compared the difference in efficacy outcome between ITT and biomarker population. For the venlafaxine study, we did not observe significant differences between populations in treatment effects at week 10 (Supplementary Table 4) with any of the rating scales. For the paroxetine study, the biomarker subpopulation had a higher response in the MADRS and Bech scores (Supplementary Table 4), whilst there was no difference between the treatment effects observed by the HAMD and IDS scales. Since we observed a slight but significant difference in IDS at baseline between biomarker population and ITT in the venlafaxine study (not shown) and no baseline differences in HAMD, we decided to select the latter as the primary scale for the biomarker analyses in the two studies.

For categorical analysis, patients were classified as responders and non-responders at week 10 based on the total HAMD score. A HAMD responder was defined as a subject who had a ≥50% reduction from baseline in HAMD total score.

In the paroxetine study, of 52 patients in the paroxetine treatment group, 36 (69%) showed a reduction in HAMD scores of at least 50% at the end of the 10-week treatment period. The remaining 16 (31%) were non-responders to paroxetine treatment. Of 54 patients in the placebo treatment group, 19 (35%) showed a reduction in HAMD scores of at least 50% at the end of the 10-week treatment period. In the venlafaxine study, of 51 patients in the venlafaxine treatment group, 36 (71%) showed a reduction in HAMD scores of at least 50% at the end of the 10-week treatment period, whereas 15 (29%) were non-responders. Of 53 patients in the placebo treatment group, 29 (55%) showed a reduction in HAMD scores of at least 50% at the end of the 10-week treatment period. At baseline, the severity of depression based on HAMD did not importantly differ between responder and non-responder groups.

Finally, to address potential gender effect, we tested for differences in response between males and females in the biomarker population, by looking at drug treatment and placebo arms separately. No significant differences were observed neither in the paroxetine nor in the venlafaxine study (see Supplementary Table 4).

### Data handling and normalisation

Since the biomarker distribution appeared to be skewed, log-transformation was applied. Therefore, all plots and analyses are provided on the log-scale.

For TNF-α and IL-10, a large number of samples were below the limit of quantitation (BLQ) (Supplementary Table 5). Data below the official LQ value were still considered in the statistical analysis in order to avoid missing any potential information available. For TNF-α and IL-10, given the amount of data below the LQ, left censoring models (adjusted for gender and centre) and non-parametric tests (Wilcoxon) were used to confirm the results from standard analyses of covariance. All models provided very similar results. We then tested for difference in biomarker baseline values between females and males in the two studies. We observed some small, but significant, differences for PAI-1, MMP9 and TNFRII (see Supplementary Fig. 2), further supporting our choice to account for gender in our subsequent statistical analyses.

### Correlation between biomarker levels and severity of depression at baseline

The first objective of this study was investigating the association between biomarker levels and MDD severity before treatment, with the aim of identifying state biomarkers.

In the paroxetine study, at randomisation we observed a positive correlation between IL-6 (*r* = 0.23, *p* = 0.018) and IL-10 (*r* = 0.19, *p* = 0.045) levels and severity of depression evaluated by HAMD. A negative correlation trend was seen for TNFRII (*r* = −0.17, *p* = 0.076). In order to identify possible gender-related differential responses, the analysis of the association of cytokine levels with HAMD was conducted separately for gender. At baseline, the positive correlation found for IL-6 in the full population was still present in males (*r* = 0.35, *p* = 0.034). In addition, significant negative correlations were found for TNFRII (*r* = −0.44, *p* = 0.004) and CRP (*r* = −0.34, *p* = 0.041), respectively. Trend to correlation could be observed for TNF-α (*r* = 0.30, *p* = 0.075). Remarkably, no associations were found in females, except for a correlation trend in MMP9 (*r* = −0.22, *p* = 0.055).

In the venlafaxine study, at randomisation, we observed a trend for positive correlation between BDNF levels (*r* = 0.19, *p* = 0.068) and severity of depression evaluated by HAMD. No correlations were detected for the other biomarkers. Also for the venlafaxine study, the analysis of the association of biomarker levels with HAMD was conducted separately for gender. At baseline, the trend for positive correlation for BDNF levels found in the full population was significant in females (*r* = 0.35, *p* = 0.003), whereas no significant correlations were found in males.

### Correlation between biomarker changes and clinical endpoint

Next, we analysed the association between changes in blood biomarkers and changes in depressive symptoms, in order to identify biomarkers of treatment efficacy.

In the paroxetine study, the statistical analysis revealed a number of significant correlations between changes in biomarker at week 10 from baseline levels and changes in HAMD total score in the same time interval. Increase in biomarker levels after treatment was significantly correlated with reduction in depression symptomatology for TNF-α (*r* = −0.22, *p* = 0.020), IL-6 (*r* = −0.23, *p* = 0.016), IL-10 (*r* = −0.23, *p* = 0.022) and CRP (*r* = −0.30, *p* < 0.001) (Table 2), where the negative signs indicate a correlation between biomarker increase and HAMD score reduction (week 10 vs. baseline). However, in the venlafaxine study, with the exception of a trend for correlation between PAI1-active level increase and HAMD reduction, the correlations between change from baseline level of the biomarkers and changes in HAMD score did not reach significance (Table 2).

**Table 2:** Correlations between changes in biomarkers at week 10 from baseline and changes in HAM-D total score at week 10 from baseline (full population, males and females separately)

Biomarker	Paroxetine			Venlafaxine		
	Full population	Males	Females	Full population	Males	Females
TNF-α	**−0.22 (p** **=** **0.020)**	*−0.31 (p* *=* *0.078)*	−0.13 (p = 0.271)	0.05 (p = 0.627)	−0.22 (p = 0.220)	0.21 (p = 0.107)
IL-6	**−0.23 (p** **=** **0.016)**	−0.23 (p = 0.211)	*−0.22 (p* *=* *0.063)*	0.03 (p = 0.754)	**−0.37 (p** **=** **0.021)**	**0.25 (p** **=** **0.049)**
IL-10	**−0.23 (p** **=** **0.022)**	**−0.40 (p** **=** **0.015)**	−0.16 (p = 0.185)	−0.15 (p = 0.131	−0.15 (p = 0.414)	−0.09 (p = 0.486)
PAI1active	−0.07 (p = 0.481)	−0.25 (p = 0.155)	0.03 (p = 0.834)	*−0.19 (p* *=* *0.055)*	−0.15 (p = 0.381)	**0.35 (p** **=** **0.003)**
BDNF	−0.13 (p = 0.219)	−0.09 (p = 0.631)	*−0.21 (p* *=* *0.080)*	0.08 (p = 0.422)	−0.07 (p = 0.692)	0.15 (p = 0.268)
MMP9	−0.02 (p = 0.867)	−0.17 (p = 0.373)	0.06 (p = 0.605)	0.15 (p = 0.148)	−0.01 (p = 0.955)	**0.27 (p** **=** **0.029)**
TNFRII	−0.14 (p = 0.159)	−0.12 (p = 0.515)	−0.16 (p = 0.183)	−0.16 (p = 0.113)	**−0.39 (p** **=** **0.013)**	−0.08 (p = 0.541)
CRP	**−0.30 (p** **<** **0.001)**	**−0.44 (p** **=** **0.004)**	**−0.24 (p** **=** **0.040)**	−0.02 (p = 0.822)	−0.03 (p = 0.888)	−0.03 (p = 0.825)

Bivariate mixed model analyses with change in biomarker and change in HAM-D as dependent variables, adjusting for centre, biomarker baseline and HAM-D baseline. The negative signs indicate a correlation between increase in biomarker and decrease in HAMD, when considering w10 vs baseline

Bold: p < 0.05; italics: 0.05 < p < 0.1

We also observed a gender effect (Table 2). In the paroxetine study, the correlation with HAMD reduction was significant for change in IL-10 (*r* = −0.40, *p* = 0.015) and change in CRP (*r* = −0.44, *p* = 0.004) in male patients. Changes in TNF-α (*p* = 0.078) demonstrated trend to correlation with HAMD reduction. Female subjects showed similar but weaker correlations in changes over time. A significant correlation with HAMD reduction was observed in females for CRP (*r* = −0.24, *p* = 0.040) with a trend towards correlation for IL-6 (*p* = 0.063) and BDNF increase (*p* = 0.080). No correlations were observed for MMP9 and TNFRII either for males or for females. In the venlafaxine study, there were significant correlations for IL-6 changes with changes in HAMD score in both male and females, but in opposite directions (Table 2). In females, we observed significant correlation between HAMD reduction and reduced levels of PAI1-Active (*r* = 0.35, *p* = 0.003) and MMP-9 (*r* = 0.27, *p* = 0.029), while males showed a significant correlation between TNFRII increase and HAMD reduction (*r* = − 0.39, *p* = 0.013).

### Prediction of treatment response from baseline biomarker profile

Subsequently, we investigated the association between baseline biomarker profile and changes in efficacy endpoints, with the objective of identifying biomarkers able to predict treatment response.

In the paroxetine study, we discovered an association between higher biomarker baseline levels and better response for TNF-α and IL-10 in the paroxetine group (Table 3), which was not observed in the venlafaxine study. In the venlafaxine study, we found a significant association between baseline levels of CRP and changes in HAMD (Table 3).

**Table 3:** Significance of baseline and baseline-treatment interaction in the analysis of change in HAM-D at week 10 from paroxetine and venlafaxine studies

Paroxetine	Overall Model (Pbo + Parox)		Placebo only	Paroxetine only
Biomarker	Baseline	Baseline*Treat.	Baseline	Baseline
TNFα	p = 0.155	*p* *=* *0.085*	p = 0.842	**p** **=** **0.036**
IL-6	p = 0.567	p = 0.125	p = 0.600	p = 0.104
IL-10	p = 0.152	*p* *=* *0.054*	p = 0.869	**p** **=** **0.009**
PAI1active	p = 0.577	p = 0.834	p = 0.798	p = 0.504
BDNF	p = 0.138	p = 0.956	p = 0.467	p = 0.270
MMP9	p = 0.615	p = 0.790	p = 0.561	p = 0.557
TNFRII	p = 0.958	p = 0.324	p = 0.496	p = 0.305
CRP	p = 0.467	p = 0.278	p = 0.853	p = 0.225
**Venlafaxine**	**Overall Model (Pbo** **+** **Venla)**		**Placebo only**	**Venlafaxine only**
Biomarker	Baseline	Baseline*Treat.	Baseline	Baseline
TNFα	p = 0.893	p = 0.973	p = 0.948	p = 0.745
IL-6	p = 0.944	p = 0.574	p = 0.698	p = 0.943
IL-10	p = 0.842	p = 0.741	p = 0.958	p = 0.877
PAI1active	p = 0.309	p = 0.927	p = 0.466	p = 0.581
BDNF	p = 0.603	p = 0.511	p = 0.814	p = 0.310
MMP9	p = 0.333	p = 0.860	p = 0.646	p = 0.424
TNFRII	p = 0.742	*p* *=* *0.099*	p = 0.157	p = 0.216
CRP	**p** **=** **0.001**	p = 0.259	**p** **=** **0.005**	*p* *=* *0.078*

Analyses of covariance accounting for the effects of HAM-D baseline value, gender and centre, as well as biomarker baseline and treatment by biomarker baseline interaction

Bold: p < 0.05; italics: 0.05 < p < 0.1

**Fig. 1:**
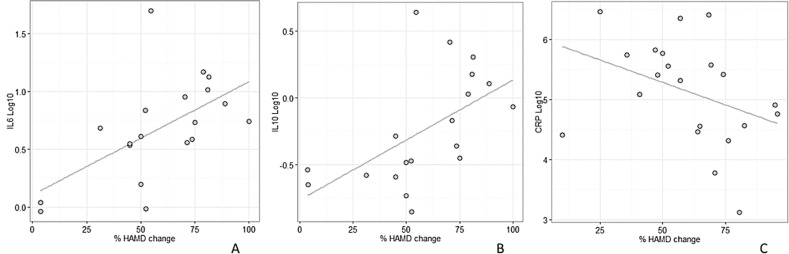
Scatterplots of baseline protein levels (log scale) vs. changes in HAMD score at week 10 for male subjects receiving paroxetine (**a**: IL-6; **b**: IL-10) or venlafaxine (**c**: CRP); the least squares linear regression line is displayed in grey

We then investigated the correlation between biomarker baseline values and HAMD score over time, separately for males and females. In the paroxetine treatment group, significant Spearman’s correlations were found in males between biomarker baseline values and larger percent changes from baseline in HAMD score over time for IL-6 (ρ = +0.67, *p* = 0.0016), and IL-10 (ρ = + 0.68, *p* = 0.0014) (Fig. 1a, b). The significant association between baseline levels of CRP and changes in HAMD detected in the venlafaxine study was found only in males (ρ = −0.45, *p* = 0.042) (Fig. 1c).

**Fig. 2:**
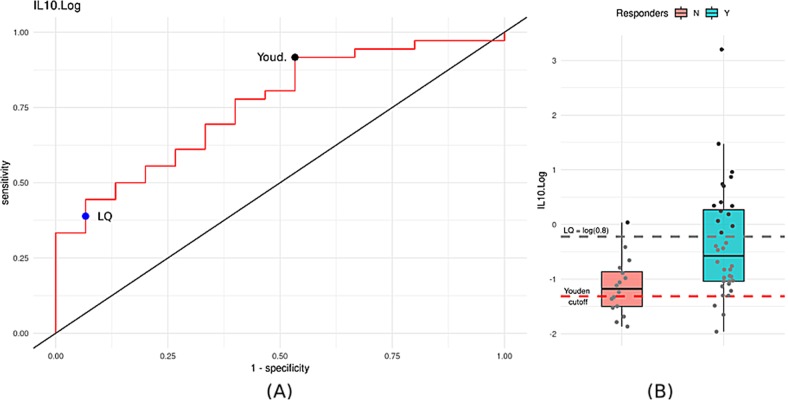
**a** Univariate ROC curve analysis for IL-10. AUC value is 0.757. The blue dot represents the BLQ cut-off of 0.8, while the black dot is the best cut-off point based on Youden’s index. **b** Distribution of IL-10 in the paroxetine study divided by responders and non-responders. Dashed red line represents the optimal Youden’s cut-off, while grey dashed line the BLQ cut-off

We then examined differences in the baseline levels of all markers between responder and non-responder groups separately for drug treatment (either paroxetine or venlafaxine) and placebo (see Supplementary Fig. 3). Significant differences between responders and non-responders at baseline levels were detected only for IL-10: the paroxetine responders showed higher baseline IL-10 levels compared to non-responders (*p* = 0.0099 based on logistic regression in the paroxetine group, see Supplementary Fig. 3), suggesting that increased IL-10 concentration at baseline may predict a good outcome from antidepressant treatment. In the placebo group, there was no association between high IL-10 baseline levels and changes in HAMD score at week 10, suggesting that this finding may be relevant to paroxetine treatment only. To provide an estimate of the predictive potential and accuracy of our markers in classifying patients according to paroxetine response status, a ROC curve analysis was performed. Figure 2 reports the results obtained for IL-10 for the prediction of paroxetine responders, which indicates an AUC = 0.757. Based on the Youden test, the best cut-off is at 0.27 pg/ml resulting with an overall accuracy of 0.78 (see Supplementary Table 6); however, this value is below the limit of quantification, and therefore unlikely to be a robust threshold. By using the LQ (less than 0.8 pg/ml) as potential “technical” cut-off criteria for prediction, we obtained a lower overall accuracy (0.55), but a much higher specificity (0.93) and Positive Predictive Value (0.93). By sub-dividing paroxetine subjects according to this BLQ value, almost all patients who had baseline IL-10 levels above 0.8 pg/ml (about 30%) showed a complete response to therapy, suggesting that subjects with increased IL-10 concentrations are potential paroxetine responders (see also Supplementary Fig. 4). Finally, we performed a PLS-DA analysis in order to assess the accuracy of the marker set as a response classifier (see Supplementary Fig. 5). The result of this analysis suggests that the predictive accuracy of the full marker set is similar to that of IL-10 as a single marker. Indeed, IL-10 appears to be the marker providing the highest contribution to the performance a classifier among all of markers of the full set.

## Discussion

This study aimed at investigating the association between the peripheral levels of eight putative biomarkers with MDD and with response to antidepressant or placebo treatment. We observed significant association with HAMD levels for IL-6, TNF-α, TNFRII, IL-10, CRP, BDNF, PAI1-active and MMP9, although these correlations were not consistently observed across studies and genders. Focussing on the most-investigated pro- and anti-inflammatory cytokines, in the paroxetine study IL-6 and IL-10 had higher baseline values in patients with higher HAMD scores, in good agreement with available evidence that MDD patients express higher levels of these cytokines^11,15^. We also found that positive changes in TNF-α, IL-6, IL-10 and CRP levels significantly correlated with higher reduction in depression symptoms. Finally, one of the most interesting findings is that baseline IL-10 levels (as well as IL-6 and TNF-α levels in male patients) could identify patient subgroups represented by subsequent responders and non-responders to paroxetine, but this was not the case for venlafaxine. IL-10 is a multifunctional cytokine playing a key role in regulating the innate and adaptive immune responses to infections and the Th1/Th2 cytokine balance^31^. Its function appears to be to limit and ultimately terminate inflammatory responses. IL-10 is produced by Th2 cells, as well as by a subset of regulatory T cells, by monocytes and dendritic cells, and can inhibit activation of pro-inflammatory cytokine production by macrophages and Th1 cells^32^. Higher levels of IL-10 have been shown in late-onset MDD^33^, which was earlier suggested to have better prognosis with respect to early-onset depression^34^. On the above premises, one might speculate that high IL-10 levels identify a sub-set of patients characterised by increased anti-inflammatory response with prevailing Th2 component. Of note, whilst IL-10 peripheral levels are reduced in mouse chronic stress models, administration of IL-10 is able to revert stress-induced depressive-like behaviours^35,36^. Similarly, IL-10 deletion in mice results with depressive-like behaviours, which are also reversed by IL-10 administration^37^.

Several groups have investigated the interactions between the clinical course of MDD, treatment conditions and immunological parameters. Recent meta-analyses of studies comparing depressed patients to healthy controls provided evidence that peripheral levels of IL-6, TNF-α, IL-10, TNFRII, and CRP were significantly higher in the MDD group, whereas IFN-γ were slightly lower^11,13,15,38^. A high degree of data heterogeneity between studies was observed, possibly due to differences in assay methods, medication status, length of disease, potential confounders such as body mass index and smoking, and endophenotypic heterogeneity of MDD^11^. A subset of studies addressed the correlation between peripheral inflammatory marker levels and MDD severity. Significant high correlations between IL1-β, IL-2, IL-4, IL-5, IL-6, IL-10, IL-12, IL-13, GM-CSF, IFN-γ and TNF-α and BDI or HAMD scores were detected in unmedicated MDD patients^38–44^. On the other hand, other studies found no significant associations between plasma or serum concentrations of several cytokines and cytokine receptors and depression severity scores^45–48^.

Several investigations have looked at antidepressants and their effect on the inflammatory response; however, again results are not consistent and a high degree of heterogeneity is observed. Nevertheless, recent meta-analyses provide overall evidence that antidepressant treatment may decrease peripheral cytokine levels^16,18–20,49,50^. In particular, stronger evidence is available for IL-6, TNF-α, IL-10 and CCL-2^20^. The correlation with the reduction of depression symptomatology we observed for TNF-α, IL-6, and IL-10 levels is not fully consistent with the reported alterations of these cytokines after antidepressant treatment according to most recent meta-analyses^16,20^. However, these inconsistencies could be justified by the heterogeneity of the studies in terms of classes of antidepressants. Based on the complexity of the interaction between MDD, antidepressants and the immune system, a broader quantification of the Th1/Th2 cytokines balance would be required^51^.

The correlation between baseline biomarker levels and the response to antidepressant treatment was also investigated. Cattaneo et al. assessed the leukocyte mRNA expression of inflammatory-related genes in antidepressant-treated patients, showing that higher levels of the three inflammation-related genes (IL-1β, MIF, and TNF-α) could predict lack of response to antidepressants^52^. The same group was able to identify an absolute cut-off level of IL-1β, MIF mRNA for prediction of non-response on an absolute basis^53^. Higher baseline plasma IL-6, TNF-α and CRP proteins have been reported in non-responders to antidepressant treatment^22,25,43,47,48,54^, even though in other studies the opposite was found for IL-6^55^ and TNF-α^39^. Other studies showed no association between baseline IL-6, IFN-γ, TNF-α, IL-10 and CRP levels and treatment response^22,54,56–58^. A meta-analysis of the data suggested overall no significant differences in levels of baseline TNF-α, IL-6 and CRP between patients subsequently responding or not responding to antidepressant treatment, although with large heterogeneity^18^.

We discovered higher IL-10 values in responders; in particular, high IL-10 levels could predict response to paroxetine treatment. However, the same pattern could not be reproduced in the venlafaxine study. These findings suggest that predictive biomarkers specific for each antidepressant class will need to be identified in order to be able to effectively predict efficacy. The difference in biomarker effects we observed between paroxetine and venlafaxine might be explained by different mechanisms of action, and in particular, different effects on the inflammatory pathway and on the Th1/Th2 balance^51^. In partial agreement with our results, Chen et al.^59^ discovered that a different peripheral cytokine response was elicited by paroxetine vs. venlafaxine treatments in MDD patients, suggesting that these antidepressants have different immunomodulatory properties. Moreover, recent findings show that different baseline immune-inflammatory biomarkers are associated with response to different antidepressant treatments, in agreement with the present study, implying the possibility of matching the appropriate therapy to a given patient based on baseline biomarkers^60,61^.

An additional finding of our study was the difference in male and female biomarker profiles, and in particular, pro- and anti-inflammatory cytokine profiles, which were found to correlate both with depression severity and with antidepressant response. Effects of gender on cytokines and other circulating biomarker levels have not been extensively studied in MDD. Because the immune response in women can be affected by oestrogen, it would be necessary to control for menstrual phases and oral contraceptive use to precisely evaluate the gender differences in cytokine production. The observation that women experience higher rates of depression than men, and also show higher rates of autoimmune diseases, has suggested inflammation as a key contributor to MDD especially for women^62^. However, available evidence supports the notion of gender-related differential responses, more than elevated inflammation specifically apparent in depressed women. Consistent with our results, Penninx et al.^63^ observed a significant gender difference in the association between depressed mood and IL-6 levels, with a stronger effect detected in men. Moreover, in an epidemiological study in psychiatric patients, it was reported that age and gender significantly affect plasma cytokine levels^64^. Kim et al.^42^ have previously reported gender differences in the levels of stimulated cytokine production in MDD patients: IL-6, TNF-α, and IFN-γ levels were significantly lower in female patients at baseline and after antidepressant treatment. Myin et al. found gender differences in changes of plasma IFN-γ levels after antidepressant therapy^65^. In a general population-based sample, significantly higher IL-1Ra and lower IL-1β levels were detected in males with depressive symptomatology, and not in females^66^. Other population studies showed that higher CRP levels^67–69^ and higher IL-6 levels^69^ were associated to higher levels of depressive symptoms specifically in men. In adolescent patients, Pallavi et al.^70^ discovered that only females had higher IL-6 as compared to the respective healthy controls. More recently, Majd et al.^71^ investigated gender difference in response in stimulated cytokine production from depressed patients. Interestingly, the study showed a significant positive association between depressive symptoms and stimulated TNF-α in men, whereas a negative association between depressive symptoms and stimulated TNF-α and IL-10 was observed in women. Overall, this collection of partially contradictory findings suggests that further investigations are needed to address gender-related differences in peripheral biomarkers.

The above literature findings share a reasonable consistency with our results. However, there are a number of conflicting findings with the literature, and within literature data. It should be noticed that some markers previously associated with depression severity or with antidepressant response outcome, such as BDNF^3^, did not show any association with treatment response in our study. Differences in the study design, in the clinical population and in biomarker assays may explain this lack of replication. In addition, literature findings mainly suggest a negative treatment outcome based on baseline elevation of pro-inflammatory cytokines, whilst our study indicates a wider activation of immunological pathways in responders, including pro- and anti-inflammatory cytokines, with a predominant and facilitating role of IL-10 in the response to paroxetine. A possible explanation for different results may lie in the differences in sampling schedules and assay procedures. In our investigations, we used a plasma assay for cytokine concentration, whereas other groups measured cytokine concentrations from isolated (and stimulated) blood cells or in serum. Plasma samples have the advantage of determining cytokine production of leukocytes in a more physiological setting, and are not affected by coagulation-induced interferences and technical issues related to fibrin formation typical of serum. Artifactual changes in blood analytes may prevent accurate diagnosis of disease states or other physiological conditions. Some changes appear to occur rapidly in serum, even when the blood is handled in a manner deemed appropriate by NCCLS. Although proteins are the ultimate effectors of most cellular processes, It should be pointed out that investigations of cytokine mRNA levels appear to be more consistent across studies, and they have resulted with more reliable and accurate predictors of antidepressant response with respect to protein levels^24,53,72^. The same applies to BDNF, for which studies on peripheral blood mRNA have resulted with more consistent results with respect to serum or protein level analyses^73^. Indeed, mRNA profiles of peripheral blood cells might be less dependable on analytical factors and they could serve as a good proxy for CNS expression^74^. As observed by other investigators^11^, irrespective from the modality, the lack of complete agreement between findings from different studies may be further explained by the fact that MDD is a heterogeneous disorder, and different subtypes may have different physiological profiles.

A limitation of our investigation is the lack of control for potential confounders such as cigarette smoking, BMI, comorbid diseases, or socioeconomic status. In addition, most of the markers tested could be affected by concomitant inflammatory conditions or by anti-inflammatory treatment. Furthermore, samples were not run with replicates, and the results here reported are not adjusted for multiple testing and might be partly false positives. An additional limitation of the present study is that strict inclusion criteria were applied^29^. Therefore, it is to be expected that the population characteristics do not mirror the wider clinical population of MDD patients and the external validity/translatability to the very heterogeneous MDD population at large may be partial. Moreover, a relatively small patient number was investigated in each study, thus replication in independent samples will be needed to validate our findings. Also, the applicability to other SSRIs or to antidepressants with different mechanism of action will need to be further tested and validated in additional clinical trials.

In conclusion, our study suggests that the assessment of a combination of anti- and pro-inflammatory cytokines may be valuable for informing response to antidepressant therapy, as previously proposed^52,60^. Our data indicate that increased baseline levels of IL-10, IL-6 and TNF-α in MDD patients may be associated with better response to treatment with paroxetine. Therefore, immune dysregulation in depressive disorders does not involve exclusively pro-inflammatory cytokines, such as IL-6 and TNF-α, but also activation of anti-inflammatory cytokines, which may result in positive treatment outcomes. In addition, we have shown a different pattern of immune system activation during depression and antidepressant treatment in male and female patients.

Although the above data are preliminary, the potential for IL-10, IL-6 and TNF-α as biomarkers for paroxetine response warrants further investigation and replication in additional trials.

## Supplementary information

Supplementary Tables

Supplementary Figures
