# Supplementary material for: Biomarkers for response in major depression: comparing paroxetine and venlafaxine from two randomised placebo-controlled clinical studies
Source: Transl Psychiatry. 2019 Aug 2;9:182. doi: 10.1038/s41398-019-0521-7 (PMC6677721; doi:10.1038/s41398-019-0521-7)
Supplement: Supplementary file 1 — Supplementary Tables [file 41398_2019_521_MOESM1_ESM.pdf]

Supplementary Table 1: Candidate biomarkers

Biomarker	*Biological function*
IL-6	Pro-inflammatory cytokine
TNF-α	Pro-inflammatory cytokine
TNFRII	Pro-inflammatory cytokine receptor
IL-10	Anti-inflammatory cytokine
CRP	Acute inflammatory phase protein
BDNF	Brain-derived neurotrophic factor
PAI1active	Inhibitor of Tissue Plasminogen Activator
MMP9	Matrix metallo protease (extracellular matrix)

Supplementary Table 2

Standard curve precision profiles and control performances of the analytical method for biomarker assessment.

Biomarker	Pg/ml	Average %CV	Biomarker	Pg/ml	Average %CV
BDNF	3200	4	IL10	400	3.9
	1600	3.6		200	6.5
	400	7.5		50	6.2
	100	9.5		125	6.6
	25	10.3		3.1	7.1
	6.25	8.8		0.8	4.5
	3.13	9.4		0.4	6.8
	0	9.7		0	8.7
MMP9	50000	0.9	IL6	200	4.3
	25000	4.2		100	6.5
	6250	4		25	5.1
	1562.5	7.5		6.3	3.3
	390.6	6.8		1.6	3
	97.7	4		0.4	7.6
	48.8	2		0.2	5.3
	0	3.5		0	7
TNFRII	400	3.6	TNFa	2400	4.8
	200	4.2		1200	3.5
	50	3.6		300	5.4
	12.5	4.5		75	4.6
	3.1	4.3		18.8	3.8
	0.8	6.2		4.7	3.3
	0.4	6.5		2.3	6.9
	0	4.4		0	8
CRP	600	2.7	PAI1a	8000	5.4
	300	4.1		4000	3.4
	75	3.8		1000	8.1
	18.8	5.6		250	4.4
	4.7	3.3		62.5	8.7
	1.2	2.7		15.63	8.7
	0.6	3.5		7.81	8.5
	0	4.2		0	11.5

Precision Profiles

Analyte		TNFa	IL6	IL10	PAI1a	BDNF	MMP9	TNFRII	CRP
Control 1	**Average**	379.3	36.7	71	498.6	309.2	4082.4	30.5	29.3
	1SD	20.2	7.2	11.7	111.9	86.2	1183.1	6.8	7.9
	%CV	5.3	19.7	16.5	22.4	27.9	29	22.4	27.1
	SD2 +	419.6	51.2	94.5	722.3	481.5	6448.6	44.1	45.2
	SD02 -	338.9	22.2	47.6	274.9	136.9	1716.1	16.8	13.4
Control 2	**Average**	86.1	6.6	16.1	96	56.3	678.3	4.2	5.9
	1SD	14.9	0.8	1	27.3	19.1	182.5	0.7	1.6
	%CV	17.3	12.3	6.2	28.4	33.9	26.9	17.1	26.5
	SD2 +	115.9	8.2	18.1	150.6	94.6	1043.3	5.6	9.1
	SD02 -	56.2	5	14.1	41.4	18.1	313.4	2.7	2.8

Control Performance

TNFa	IL6	IL10	PAI1a	BDNF	MMP9	TNFRII	CRP
4.7 pg/ml	0.4 pg/ml	0.8 pg/ml	195.9 pg/ml	156.5 pg/ml	2.44 ng/ml	19.5 pg/ml	2.93 ng/ml

Lower Limit of Quantification

Supplementary Table 3

Supplementary Table 3a: Comparison of baseline demographic and clinical data of biomarker population between biomarker population and ITT population in the paroxetine study (SND103288)

		Biomarker population			ITT population		
		Paroxetine (n=52)	Placebo (n=54)	Total (n=106)	Paroxetine (n=166)	Placebo (n=156)	Total (n=322)
Age	Mean	45.88	46.35	46.12	44.40	42.90	43.1
	SD	9.99	9.69	9.84	10.90	10.99	10.96
	Min	22	21	21	20	19	19
	Max	63	63	63	64	64	64
Sex	Female	33 (63%)	39 (72%)	72 (68%)	111 (67%)	117 (75%)	228 (71%)
	Male	19 (37%)	15 (28%)	34 (32%)	55 (33%)	39 (25%)	94 (29%)
HAM-D	Mean	21.67	23.40	22.56	23.5	22.4	22.9
	SD	3.84	4.34	4.19	4.64	4.65	4.67
	Min	13	10	10	10	13	10
	Max	32	32	32	32	37	37

Supplementary Table 3b: Comparison of baseline demographic and clinical data of biomarker population between biomarker population and ITT population in the venlafaxine study (SND103285)

		Biomarker population			ITT population		
		Venlafaxine (N=51)	Placebo (N=53)	Total (N=104)	Venlafaxine (N=133)	Placebo (N=126)	Total (N=259)
Age	Mean	44.80	44.53	44.66	43.0	41.9	42.4
	SD	11.10	10.46	10.78	11.19	11.75	11.48
	Min	21	19	19	19	18	18
	Max	63	60	63	63	63	63
Sex	Female	29 (57%)	35 (66%)	64 (62%)	81 (61%)	80 (63%)	161 (62%)
	Male	22 (43%)	18 (34%)	40 (38%)	52 (39%)	46 (37%)	98 (38%)
HAM-D	Mean	23.62	24.45	24.05	23.16	23.90	23.52
	SD	4.16	4.20	4.20	4.79	5.33	5.07
	Min	16	16	16	12	11	11
	Max	34	33	34	34	37	37

Supplementary Table 4

Treatment differences for efficacy endpoints in paroxetine (SND103288) and venlafaxine (SND103285) studies

	**Treatment Difference at W10 (paroxetine – placebo)**		
Efficacy endpoint	ITT population (N=322)	Biomarker population (N=106)	Treatment*population interaction
HAMD-17	-2.96 (-4.28, -1.64)	-4.38 (-6.38, -2.38)	p=0.49
Bech	-1.77 (-2.49, -1.05)	-2.42 (-3.56, -1.29)	**P=0.09**
IDS-CR	-5.12 (-7.55, -2.68)	-7.29 (-11.11, -3.47)	P=0.36
MADRS	-3.99 (-5.75, -2.22)	-6.23 (-8.81, -3.66)	**P=0.03**
	**Treatment Difference at W10 (venlafaxine–placebo)**		
Efficacy endpoint	ITT population (N=259)	Biomarker population (N=104)	Treatment*population interaction
HAMD-17	-2.35 (-3.84, -0.86)	-2.03 (-3.83, -0.23)	P=0.87
Bech	-1.56 (-2.42, -0.71)	-1.64 (-2.75, -0.54)	P=0.96
IDS-CR	-4.45 (-7.26, -1.64)	-5.35 (-9.00, -1.70)	P=0.37

Treatment*population interaction was estimated from mixed model repeated measures analysis adjusted for centre, gender, visit and baseline*visit

Changes in HAMD-17 in the biomarker populations according to gender (Mann-Whitney U test)

HAMD W10-Baseline	Females		Males		p value MW -U Test
	Avg	SD	Avg	SD	
Paroxetine	-12.00	4.10	-13.11	7.81	0.593
Placebo (P)	-7.77	6.02	-11.47	8.87	0.106
Venlafaxine	-15.03	6.18	-14.52	5.30	0.679
Placebo (V)	-13.17	7.13	-11.94	7.07	0.618

Supplementary Table 5

Number of samples below limit of quantitation values by biomarker and visit

Biomarker	Baseline	Week 10	Total
TNFα	26 (17%)	25 (12%)	61 (15%)
IL6	1 (<1%)	0	1 (<1%)
IL10	104 (50%)	96 (46%)	200 (48%)
TNFRII	0	1 (<1%)	1 (<1%)
CRP	0	1 (<1%)	1 (<1%)

Supplementary Table 6

ROC analysis

Bio marker	AUC	accuracy	npv	ppv	sensit	specif	Threshold (pg/ml)	Threshold log	method
BDNF	0.58704	0.67	0.44	0.77	0.75	0.47	11180.41	9.32	youden
CRP	0.48519	0.47	0.33	0.80	0.33	0.80	369687.21	12.82	youden
IL10	0.75741	0.78	0.70	0.80	0.92	0.47	0.27	-1.32	youden
IL6	0.64630	0.63	0.43	0.87	0.56	0.80	5.41	1.69	youden
MMP9	0.50370	0.45	0.33	0.83	0.28	0.87	129253.01	11.77	youden
PAI1a	0.67963	0.61	0.42	0.94	0.47	0.93	8526.47	9.05	youden
TNFaa	0.54259	0.63	0.39	0.76	0.69	0.47	6.91	1.93	youden
TNFRII	0.61111	0.80	0.78	0.81	0.94	0.47	411.81	6.02	youden
BDNF	0.58704	0.67	0.44	0.77	0.75	0.47	11180.41	9.32	topleft
CRP	0.48519	0.53	0.32	0.73	0.53	0.53	149722.67	11.92	topleft
IL10	0.75741	0.69	0.48	0.83	0.69	0.67	0.38	-0.98	topleft
IL6	0.64630	0.63	0.43	0.87	0.56	0.80	5.41	1.69	topleft
MMP9	0.50370	0.53	0.32	0.73	0.53	0.53	82666.58	11.32	topleft
PAI1a	0.67963	0.67	0.45	0.81	0.69	0.60	3285.74	8.10	topleft
TNFa	0.54259	0.63	0.39	0.76	0.69	0.47	6.91	1.93	topleft
TNFRII	0.61111	0.69	0.47	0.79	0.75	0.53	525.59	6.26	topleft

Table: ROC analysis on paroxetine data for HAMD responder/non-responder analysis using log transformed biomarkers. IL10 is the biomarker with the highest accuracy for both Youden and top-left method for optimal cut-off identification.
