# Supplementary material for: Biomarkers for response in major depression: comparing paroxetine and venlafaxine from two randomised placebo-controlled clinical studies
Source: Transl Psychiatry. 2019 Aug 2;9:182. doi: 10.1038/s41398-019-0521-7 (PMC6677721; doi:10.1038/s41398-019-0521-7)
Supplement: Supplementary file 2 — Supplementary Figures [file 41398_2019_521_MOESM2_ESM.pdf]

# Supplementary Figure 1: Normality analysis

| | Raw data | | After log transformation | |
|---|---|---|---|---|
| **Biomarkers** | P value | Normality | P value | Normality |
| TNFa | 5.62E-39 | Not-Normal | 4.89E-05 | Not-Normal |
| IL6 | 3.50E-35 | Not-Normal | 2.50E-09 | Not-Normal |
| IL10 | 3.51E-38 | Not-Normal | 6.19E-10 | Not-Normal |
| PAI1a | 2.80E-29 | Not-Normal | 0.16 | Normal |
| BDNF | 2.88E-25 | Not-Normal | 0.17 | Normal |
| MMP9 | 1.51E-37 | Not-Normal | 5.09E-05 | Not-Normal |
| TNFRII | 4.63E-16 | Not-Normal | 1.26E-11 | Not-Normal |
| CRP | 4.01E-34 | Not-Normal | 0.28 | Normal |

**Table**: Shapiro normality test on each biomarker before and after log transformation. Biomarkers are normally distributed if p-value is greater than 0.05.

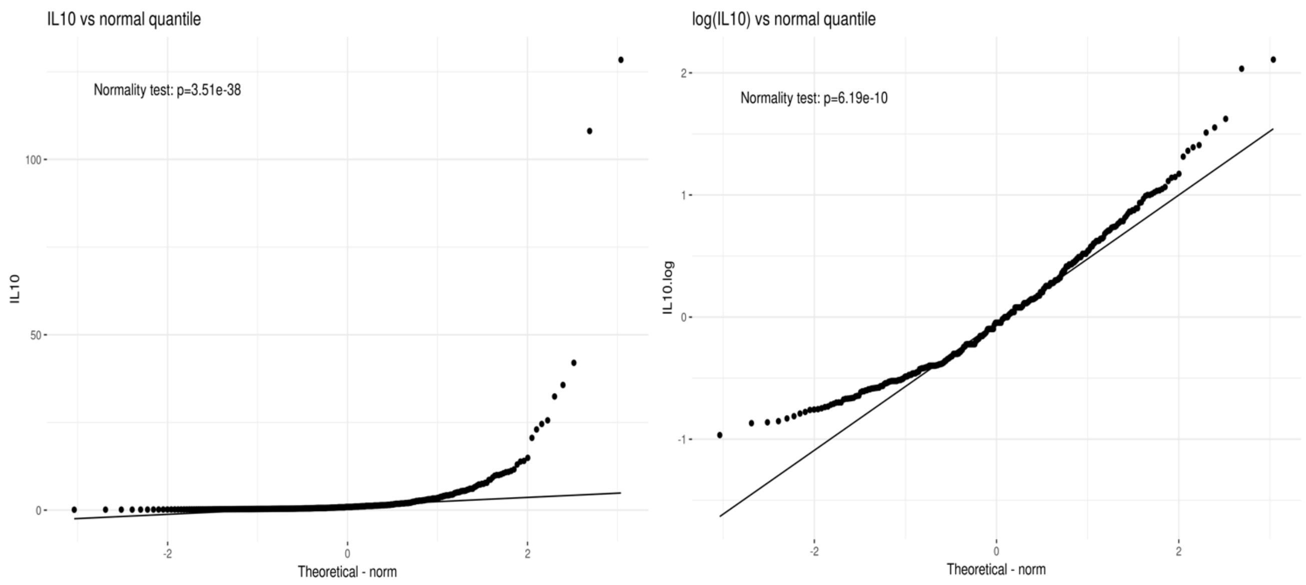


**Figure**: Distribution of quantiles of IL10 over normal distribution theoretical quantiles (QQplot) for IL10 (right) and log transformed IL10 (left).

## Supplementary Figure 2:

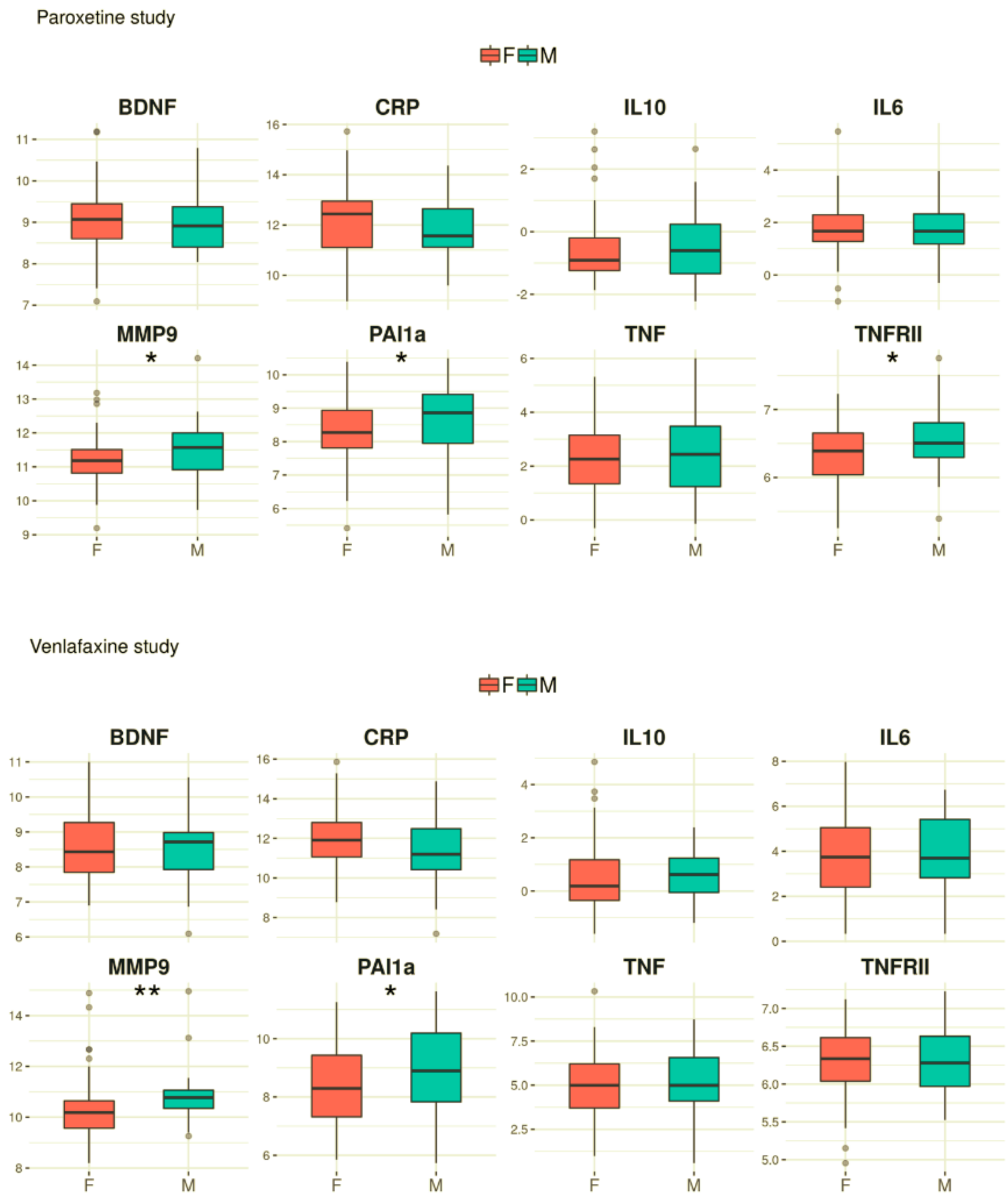


**Figure**: Distribution of biomarker levels at baseline for Paroxetine (top) and Venlafaxine (bottom) studies based on gender. shown by responders and non-responders to highlight differences between groups (Wilcoxon Test). Significant differences were found for MMP9 (p=0.04697 paroxetine, p=0.00687 venlafaxine); PAI1a (p=0.03866 paroxetine, p=0.00350 venlafaxine); TNFRII (p=0.0374 paroxetine) based on Wilcoxon Test

## Supplementary Figure 3:

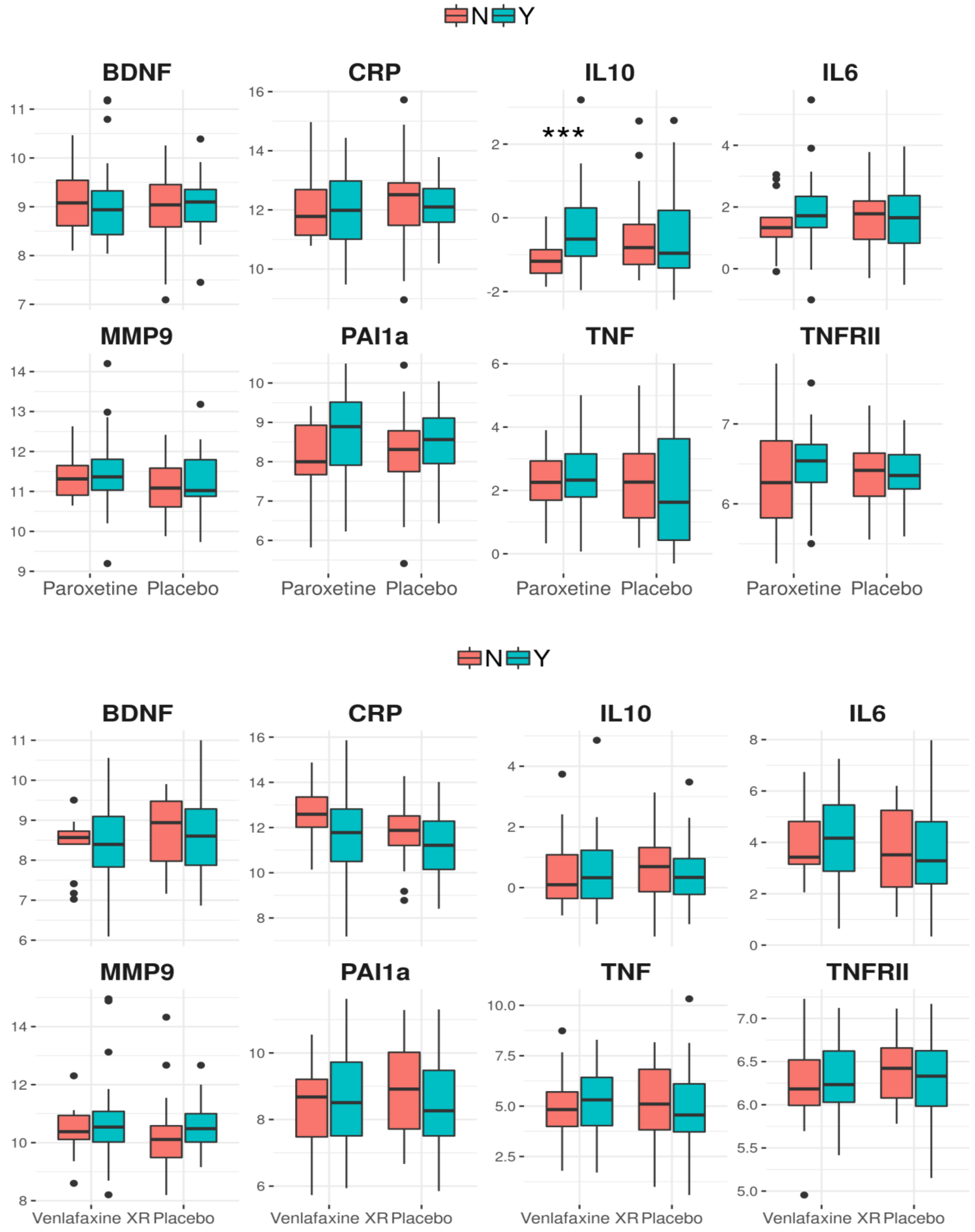


**Figure**: Distribution of biomarker levels at baseline for Paroxetine (top) and Venlafaxine (bottom) studies. Box-plots are shown by responders and non-responders to highlight differences between groups. Only IL-10 showed significant differences between the two groups (p= 0.0099) according to logistic regression.

**Supplementary Figure 4:**

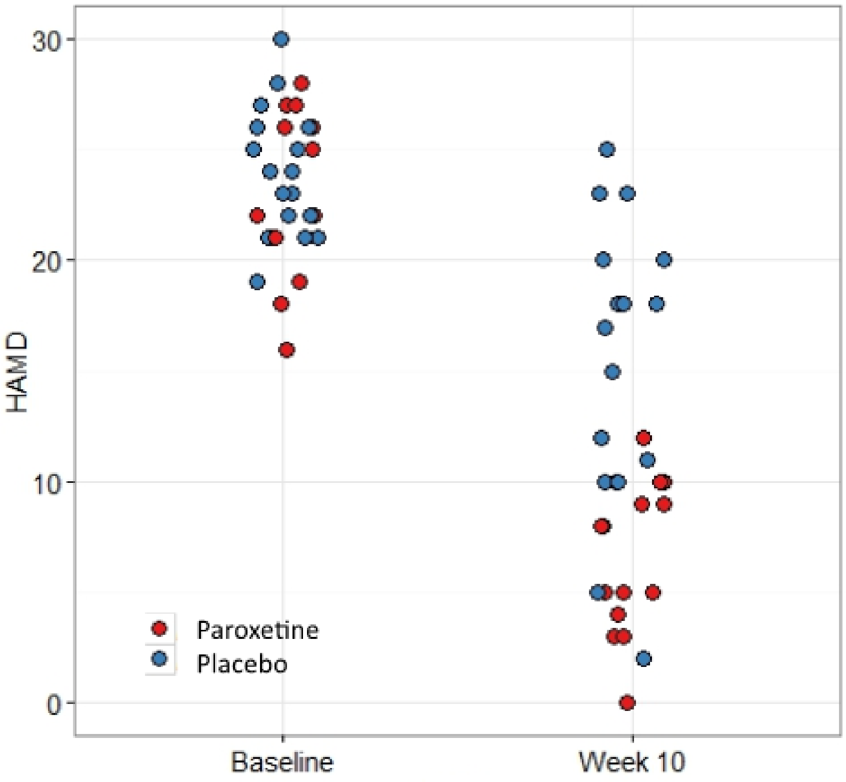


**Figure:** Pre-dose level of IL-10 was significantly higher in the responders only in the paroxetine treatment group. HAMD total score for all patients in the paroxetine study with baseline IL-10 value over BLQ (> 0.8), before and after treatment with active drug (red) or placebo (blue). All patients in the paroxetine subgroup are responders. Graphics were generated using InVivoStat (Clark et al., J Psychopharmacol 2012; 26: 1136–42).

## Supplementary Figure 5: PLS-DA

| | |
|---|---|
| Sensitivity | 0.44 |
| Specificity | 0.94 |
| Pos Pred Value | 0.78 |
| Neg Pred Value | 0.79 |
| Precision | 0.78 |
| Recall | 0.44 |
| Prevalence | 0.31 |
| Accuracy | 0.79 |
| P Value | 0.07 |

Table: PLS-DA classification measures

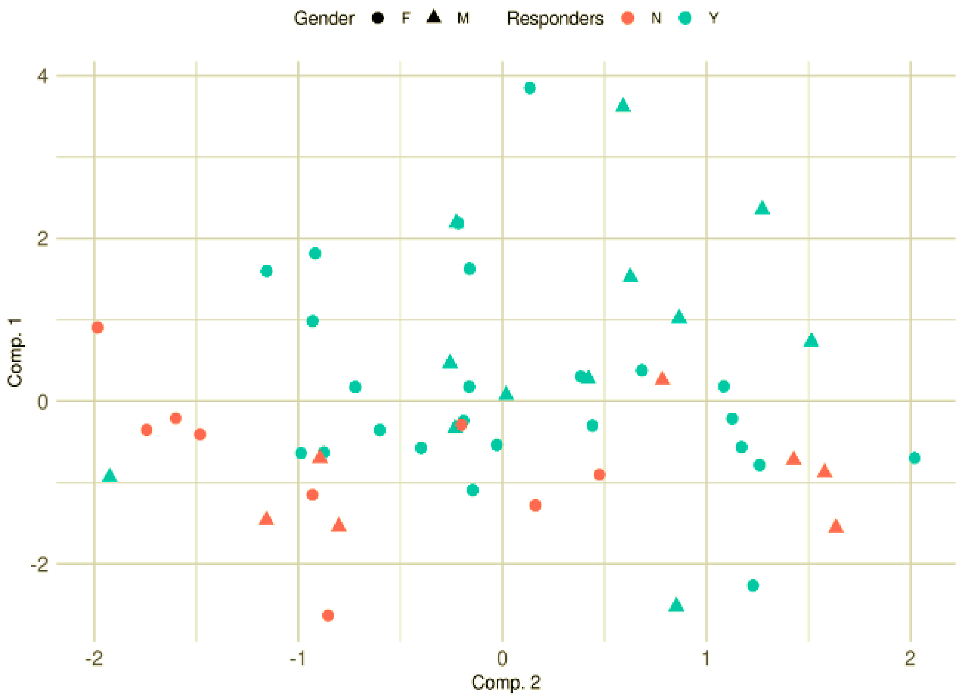


Figure: Sample distribution over first two PLS-DA components. Red dots: responders; blue dots: non-responders. Subject are divided by gender (circle for females and triangle for males). No overt effect of gender in the classification model can be observed

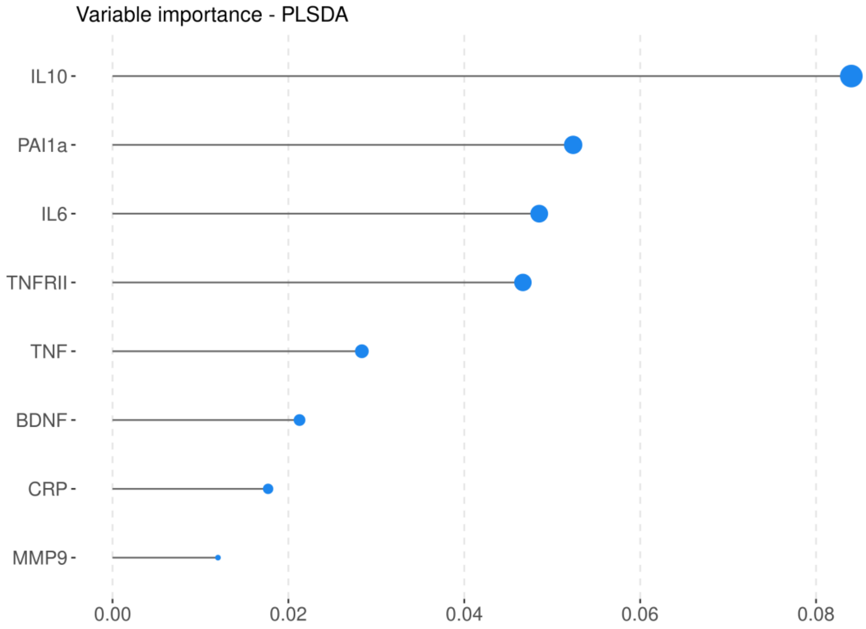


| Biomarker | VIP |
|---|---|
| IL10 | 0.084 |
| PAI1a | 0.052 |
| IL6 | 0.049 |
| TNFRII | 0.047 |
| TNFa | 0.028 |
| BDNF | 0.021 |
| CRP | 0.018 |
| MMP9 | 0.012 |

Figure: Variable importance from PLS-DA model
